# Supplementary material for: Macrophages promote endothelial-to-mesenchymal transition via MT1-MMP/TGFβ1 after myocardial infarction
Source: eLife. 2020 Oct 16;9:e57920. doi: 10.7554/eLife.57920 (PMC7609061; doi:10.7554/eLife.57920)
Supplement: Supplementary file 1. [file elife-57920-supp1.docx]

**List of primer sequences used for qPCR assays**

| **Gene** | **Sequence Forward Primer** | **Sequence Reverse Primer** |
| --- | --- | --- |
| *36b4* | GCGACCTGGAAGTCCAACTA | ATCTGCTGCATCTGCTTGG |
| *Cyclophilin* | ACAGGTCCTGGCATCTTGTC | CATGGCTTCCACAATGTTCA |
| *Mmp14* | CCCTTTTACCAGTGGATGGA | TGTCAAAGTTCCCGTCACAG |
| *Mmp2* | GTCGCCCCTAAAACAGACAA | GGTCTCGATGGTGTTCTGGT |
| *Mmp13* | ATCCTGGCCACCTTCTTCTT | TTTCTCGGAGCCTGTCAACT |
| *Mmp9* | CGTCGTGATCCCCACTTACT | AACACACAGGGTTTGCCTTC |
| *Col1a1* | GCTTCAGTGGTTTGGATGGT | AGGGCGACCTCTCTCACC |
| *Pecam* | TGCACAGTGATGCTGAACAA | CCATGAGCACAAAGTTCTCG |
| *Kdr* | GATCACCATTCATCGCCTCT | CCCAGGAAATTCTGTTTCCA |
| *Col4a2* | CAGGATTCCAAGGTGCTCAT | CTGGAAGGCCTCTCATTGAA |
| *Tagln* | GATGGAACAGGTGGCTCAAT | AACTGCCCAAAGCCATTAGA |
| *Acta2* | GAAAATGAGATGGCCACGGC | TAGGTGGTTTCGTGGATGCC |
| *Serpine1* | GTAGCACAGGCACTGCAAAA | GCCGAACCACAAAGAGAAAG |
| *Cdh5* | CAGGGAATGTGCTTGCCTAT | TCACACGGATGACAGAGGTC |
| *Tie2* | CCTTCACCAGGCTGATTGTT | ATAAACCCAGGAGGGAAAT |
| *Cdh2* | GCCATCATCGCTATCCTTCT | TTAAAAGCTGCTTGGCTTGG |
| *Col1a2* | CCAGCGAAGAACTCATACAGC | GGACACCCCTTCTACGTTGT |
| *Col3a1* | CGTAAGCACTGGTGGACAGA | AGCTGCACATCAACGACATC |
| *Zeb2* | GAAAAGCAGTTCCCTTCTGC | AGCCTCGAGTGCTCGATAAG |
| *Snai1* | TGGAAAGGCCTTCTCTAGGC | CTTCACATCCGAGTGGGTTT |
| *Il1b* | GCTGAAAGCTCTCCACCTCA | AGGCCACAGGTATTTTGTCG |
| *Il6* | CAAAGCCAGAGTCCTTCAGAG | GCCACTCCTTCTGTGACTCC |
| *Ccl2* | CCCAATGAGTAGGCTGGAGA | TCTGGACCCATTCCTTCTTG |
| *Nos2* | CAGCTGGGCTGTACAAACCTT | CATTGGAAGTGAAGCGTTTCG |
| *Tnfa* | CCAGACCCTCACACTCAGATC | CACTTGGTGGTTTGCTACGAC |
| *Cd86* | GGGCTTGGCAATCCTTATCT | CCAGCTCACTCAGGCTTATG |
| *Il12* | TGGTTTGCCATCGTTTTGCTG | ACAGGTGAGGTTCACTGTTTCT |
| *Mcp1* | AGGTCCCTGTCATGCTTCTG | GCTGCTGGTGATCCTCTTGT |
| *Arg1* | CTCCAAGCCAAAGTCCTTAGAG | AGGAGCTGTCATTAGGGACATC |
| *Chil3* | ACTTTGATGGCCTCAACCTG | AATGATTCCTGCTCCTGTGG |
| *Mrc1* | ATTGTGGAGCAGATGGAAGG | TGAATGGAAATGCACAGACG |
| *MerTK* | GACTCCCTATCCCGGAGTTC | CTGCAGCCTCAACACAGAGA |
| *Cd163* | TCTTTGTTGTGGCTGTGAGC | CCCCACTTGTCATGGATCTT |
| *Retnla* | CCCTTCTCATCTGCATCTCC | CAGTAGCAGTCATCCCAGCA |
| *Igf1* | TGGATGCTCTTCAGTTCGTG | GCAACACTCATCCACAATGC |
| *Bax* | GAAGCTGAGCGAGTGTCTCC | GAAGTTGCCATCAGCAAACA |
| *Bclxs* | CAGGGACCGCGTATCAGAGC | CAGGGACCAGCGGTTGAAG |
| *Fas* | TATCAAGGAGGCCCATTTTG | CCCCTTCTCCCAATTCTCTT |
| *Bcl2a1* | GGAGAATGGATACGGCAGAA | TTCCCAGATCTGTCCTGTCA |
| *Mmp12* | TCTGGCAATAATGCACATCC | ACATCCTCACGCTTCATGTC |
| *Cdkn1a* | TTGCACTCTGGTGTCTGAGC | TCTGCGCTTGGAGTGATAGA |
| *Scf* | TGACCTCGTGTTATGCATGG | TCAGATGCCACCATAAAGTCC |
| *Icam1* | GGGAATGTCACCAGGAATGT | GCCCACAATGACCAGCAGTA |
| *Fn1* | CATCATTTCATGCCAACCAG | CTGCAGTGCCTCCACTATGA |
| *Postn* | ATTCGAACCCGGAGTCACTA | GACGTGGATGACACCATTTG |
| *Pdgfb* | AAGTGTGAGACAATAGTGACCCC | CATGGGTGTGCTTAAACTTTCG |
| *Spp1* | GCTTGGCTTATGGACTGAGG | CGCTCTTCATGTGAGAGGTG |
| *Myh6* | TGGTCACCAACAACCCATACGACT | TGTCAGCTTGTAGACACCAGCCTT |
| *Myh7* | CCCTCCTCACATCTTCTCCA | GCGGCAATAACAGCAAAATA |
| *Tnnt3* | GGAACGCCAGAACAGATTGG | TGGAGGACAGAGCCTTTTTCTT |
| *Nppa* | ATTGACAGGATTGGAGCCCAGAGT | TGACACACCACAAGGGCTTAGGAT |
| *Pln* | TACCTCACTCGCTCGGCTAT | GATGCAGATCAGCAGCAGAC |
| *Ryr2* | CCGGTCTTCCACTGACAAAC | GAGGACACGCTGACCAAGAT |
